# Effect of endothelial progenitor cell-derived extracellular vesicles on endothelial cell ferroptosis and atherosclerotic vascular endothelial injury

**DOI:** 10.1038/s41420-021-00610-0

**Published:** 2021-09-07

**Authors:** Lin Li, Haining Wang, Jing Zhang, Xiao Chen, Zhongwang Zhang, Qiang Li

**Affiliations:** Department of Vascular Surgery, Qingdao Hiser Medical Center, 4 Renmin Road, Shibei District, 266033 Qingdao City, Shandong Province China

**Keywords:** Cell delivery, Tumour angiogenesis

## Abstract

Atherosclerosis (AS) is a chronic inflammatory disorder characterized by endothelial dysfunction. Endothelial progenitor cells (EPCs) can overcome endothelial dysfunction and reduce AS risk. This study focused on the role of EPC-secreted extracellular vesicles (EPC-EVs) in AS. First, mouse EPCs and mouse aortic endothelial cells (MAECs) were isolated and identified. EVs were isolated from EPCs and identified. EPC-EVs were co-cultured with MAECs and the internalization of EVs was observed. Glutathione (GSH) consumption, reactive oxygen species (ROS) production, lipid peroxidation, and iron accumulation and cell death in endothelial cells were detected. The binding relationship between miR-199a-3p and specificity protein 1 (SP1) was confirmed using dual-luciferase and RIP assays. The mouse model of AS was established. The relationships between miR-199a-3p expression and aortic area plaque and serum pro-inflammatory factor were analyzed. The degree of atherosclerotic lesion was detected using oil red O staining and the serum inflammatory factors were detected using ELISA. Our results elicited that EPC-EVs inhibited cell death, GSH consumption, ROS production, lipid peroxidation, and iron accumulation in endothelial cells, thereby suppressing ferroptosis of endothelial cells. EPC-EVs transferred miR-199a-3p into endothelial cells. miR-199a-3p targeted SP1. Silencing miR-199a-3p or overexpression of SP1 in endothelial cells reversed the effect of EPC-EVs on ferroptosis of endothelial cells. In vivo experiments confirmed that EPC-EVs inhibited ferroptosis of endothelial cells and then alleviated the occurrence of AS via the miR-199a-3p/SP1 axis. To conclude, EPC-EVs transferred miR-199a-3p to inhibit SP1, thus repressing ferroptosis of endothelial cells and retarding the occurrence of AS.

## Introduction

Atherosclerosis (AS), a chronic inflammatory disorder, is concerned with diverse disease states and multiple risk factors, mainly arterial hypertension, hypercholesterolemia, and diabetes [1]. AS is manifested with the pathological remodeling of arterial wall resulted from lipid accumulation in the sub-endothelial layer of the artery and the retention of lipid leads to inflammatory response [2]. The inflammatory state triggers further endothelial dysfunction and extracellular matrix remodeling, eventually resulting in the formation of calcified and vulnerable plaques [1, 3]. Since iron is a regulator of inflammatory response in the body, maintaining iron homeostasis appears to be a promising approach to prevent AS [4]. Ferroptosis represents a recently identified regulated cell death, is featured by the iron-dependent lipid hydroperoxide accumulation, which differs from other cell deaths in morphology, biochemistry, and genetics [5]. Emerging evidence has unveiled the implication of ferroptosis in the onset and progress of cardiovascular diseases (CVDs), including AS [6, 7]. Inhibition of ferroptosis alleviates AS by attenuating lipid peroxidation and alleviating endothelial dysfunction in aortic endothelial cells (ECs) [6, 8]. Exploring the underlying mechanism of ferroptosis in ECs may contribute to the further understanding on the pathological mechanism of AS and confer a novel therapeutic target for AS.

Endothelial progenitor cells (EPCs) participate in the maintenance of endothelial homoeostasis and formation of new vessels, and it is well established that AS is related to the decrease and dysfunction of EPCs [9]. EPC-based therapy is expected to cure the vessel diseases of patients with AS and diabetes, thereby providing novel therapeutic insight for the treatment of diverse CVDs [10]. Extracellular vesicles (EVs) are small membrane-bound vesicles secreted by nearly all cell types and implicated in intercellular communication [11]. EPC-derived EVs (EPC-EVs) have been demonstrated to exert considerable effects on angiogenesis and cutaneous wound healing [12]. EPC-EVs can significantly reduce atherosclerotic plaques and inflammatory cytokines and alleviate atherosclerotic endothelial dysfunction in mice [13]. Intriguingly, EPC-EVs are also reported to prevent glucocorticoid-induced osteoporosis in mice by inhibiting the ferroptotic pathway [14]. However, whether EPC-EVs can play a role in AS by inhibiting ferroptosis of ECs remains unknown.

Mechanically, EVs mediate intercellular communication by transferring bioactive molecules, such as microRNAs (miRNAs) [15]. miRNAs (~22 nucleotides in length) function as antisense RNAs to silence target genes at the post-transcription level [16], which can be exploited as novel therapeutics and clinical biomarkers of AS [17]. miRNAs regulate key lipid homeostasis pathways that alter the progression and regression of atherosclerotic plaques [18]. Importantly, the role of miRNA in cell death is also identified and a certain miRNA is reported to protect ECs from ferroptosis [19]. Nevertheless, the mechanism of EPC-EVs carrying miRNAs in AS remains largely unknown. Herein, this study investigated the mechanism of EPC-EVs carrying a miRNA in ferroptosis of ECs, which shall confer novel insight for the clinical management of AS.

## Results

### EPC-EVs could be internalized by ECs

**Fig. 1:**
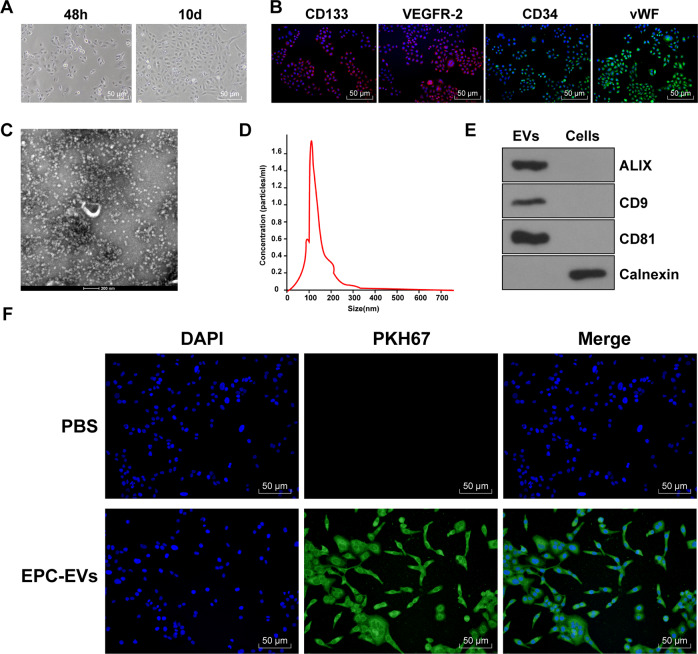
EPC-EVs could be internalized by endothelial cells. **A** The morphology of EPCs was observed under the inverted microscope (bar = 100 μm). **B** The markers of EPCs were detected using immunofluorescence (CD133 presented red and the nucleus presented blue after Hoechst3342 staining; CD34 presented green and the nucleus presented blue after Hoechst3342 staining; KDR presented red and the nucleus presented blue after Hoechst3342 staining; vWF presented green and the nucleus presented blue after Hoechst3342 staining) (bar = 50 μm). **C** TEM showed the particle diameter of EPC-EVs (bar = 200 nm). **D** NTA showed the diameter distribution and concentration of EVs. **E** The EV marker proteins Alix, CD81, CD9, and endoplasmic reticulum protein calnexin were detected using western blot. **F** PKH67-labeled EPC-EVs into ECs were observed under the immunofluorescent microscope (PKH67-labeled EPC-EVs presented green and the nucleus presented blue after Hoechst3342 staining) (scale bar = 50 μm). Each experiment was repeated three times independently.

EPCs-derived EVs can improve endothelial dysfunction in diabetic mice [13]. In this study, EPCs were isolated and purified from mouse umbilical cord blood. The morphological changes of EPCs were observed under the inverted microscope. After 48 h of seeding, some cells were attached to the wall after the first liquid exchange, and the cell body began to grow larger. The brightness of adherent cells was enhanced under the inverted microscope. On the 10th day after seeding, most cells presented short fusiform and the cell volume increased; the adherent cells nearly reached confluence, and the fusion cells formed the paving stone-like changes (Fig. 1A). Immunofluorescence assay showed that EPCs stably expressed specific antigens CD133, CD34, VEGFR-2, and vWF (Fig. 1B). EPC-EVs were further isolated from the conditioned medium of EPCs and analyzed using TEM and NTA. The results showed that the isolated EPC-EVs were bilayer vesicles, and the size of separated particles was in the range of 30–300 nm (Fig. 1C, D). Further western blot analysis showed that the expressions of ALIX, CD81, and CD9 were detected in the EVs, while non-EV marker calnexin was negative (Fig. 1E). These characteristic data indicated that EPC-EVs are successfully separated. To further explore the effect of EPC-EVs on ECs, we co-cultured PKH67-labeled EPC-EVs with ECs. After 12 h, the cells were stained and photographed (Fig. 1F). It was found that a large number of EPC-EVs entered ECs and distributed around the nucleus. These results suggested that EPCs can deliver EVs to ECs.

### EPC-EVs carrying miR-199a-3p was related to AS

**Fig. 2:**
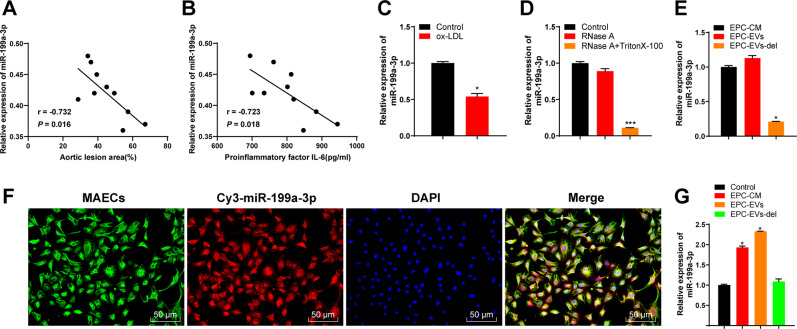
EPC-EVs carried miR-199a-3p into ECs. **A** The relationship between miR-199a-3p expression in ApoE-/- mouse aorta and the area of aortic plaque. **B** The relationship between miR-199a-3p expression in ApoE-/- mouse serum and the serum content of pro-inflammatory factor IL-6. **C** miR-199a-3p expression in ECs before and after ox-LDL treatment was detected using qRT-PCR. **D** After EVs were treated with RNase A and detergent, miR-199a-3p expression was detected using qRT-PCR. **E** miR-199a-3p expression in EPC medium (EPC-CM) and EVs (EPC-EVs/-del) was detected using qRT-PCR. **F** EPC-EVs labeled with fluorescent Cy3-miR-199a-3p into endothelial cells were observed under the confocal laser microscope (Cy3-miR-199a-3p-labeled EVs presented red and the nucleus presented blue after DAPI staining; phalloidin-labeled endothelial cells presented green) (scale bar = 50 μm). **G** After ECs were co-cultured with EPC-EVs, miR-199a-3p expression in MAECs was detected using qRT-PCR. Each experiment was repeated three times independently. Measurement data are depicted as mean ± SD. The *t* test was used for the comparisons between two groups. One-way ANOVA was employed for the comparisons among multiple groups, followed by Tukey’s multiple comparisons test. *p < 0.05.

We found that miR-199a-3p exists in a variety of EVs through EVmiRNA website (http://bioinfo.life.hust.edu.cn/EVmiRNA#!/), and EPC-derived EVs contain high miR-199a-3p expression [13]. miR-199a-3p may have a protective effect on vascular endothelium [20]. We randomly selected 10 atherosclerotic mice. Quantitative real-time polymerase chain reaction (qRT-PCR) was conducted to detect the relationship between miR-199a-3p expression in aorta and serum and the lesion area of aorta and the serum content of pro-inflammatory factor IL-6. miR-199a-3p expression was declined with the increase of the lesion area of aortic plaque and the serum content of IL-6 (Fig. 2A, B). After ox-LDL treatment in ECs in vitro, the expression of miR-199a-3p in ECs was notably decreased (Fig. 2C). Briefly, miR-199a-3p expression was related to AS.

Therefore, to further study whether EPC-EVs affected AS by delivering miR-199a-3p, we first evaluated whether miR-199a-3p existed in EPC-EVs and the effect of EPC-EVs carrying miR-199a-3p on ECs. We conducted RNase A protection assay on EVs to verify whether miR-199a-3p was encapsulated in EVs or appeared on the surface of EVs. After RNase A treatment, miR-199a-3p expression in the medium did not change, while after RNase A and Triton 100 treatment, miR-199a-3p expression was significantly reduced, indicating that miR-199a-3p was encapsulated in EVs rather than directly released (Fig. 2D). In addition, miR-199a-3p expression in EPC-EVs was equal to that in EPC culture medium, but miR-199a-3p expression almost disappeared after the EVs were removed from the culture medium (Fig. 2E), indicating that EVs were the main carrier of miR-199a-3p. Then, EPCs were transfected with red fluorescent Cy3-labeled miR-199a-3p. After 48 h, the cell supernatant was collected and ultracentrifuged to obtain EVs. EVs were co-cultured with ECs for 12 h and then stained and photographed (Fig. 2F). It was found that Cy3-labeled miR-199a-3p existed in the cytoplasm of ECs. Moreover, we found that miR-199a-3p expression in the co-cultured ECs was higher than that in the control cells, while there was no significant difference in miR-199a-3p expression in ECs cultured with EPC medium free of EVs (Fig. 2G). These results suggested that EPCs delivered miR-199a-3 to ECs through EVs.

### EPC-EVs inhibited ferroptosis of ECs through miR-199a-3p

**Fig. 3:**
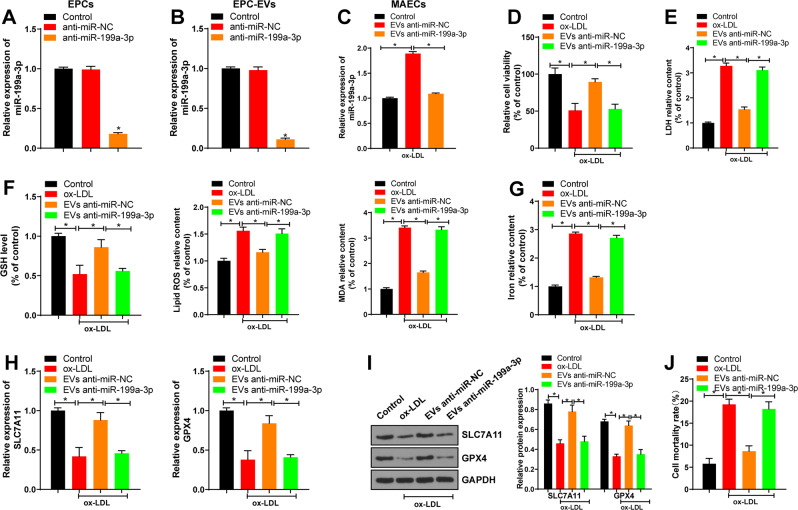
EPC-EVs inhibited ferroptosis of ECs through miR-199a-3p. MAECs were isolated from the aorta of C57BL/6J mice and treated with ox-LDL alone or in combination with EVs/EVs-anti miR-199a-3p for 24 h. **A** The efficiency of lentivirus infection was confirmed using qRT-PCR. **B** miR-199a-3p expression in EPC-EVs after lentivirus infection was detected using qRT-PCR. **C** miR-199a-3p expression in MAECs co-cultured with EVs/EVs-anti miR-199a-3p was detected using qRT-PCR. **D** The viability of MAECs co-cultured with EVs/EVs-anti miR-199a-3p was detected using CCK8 assay. **E** The cell damage was measured using LDH kit. **F** GSH consumption, lipid ROS production, and lipid peroxidation were detected using the kits. **G** The relative content of iron in MAECs was determined using the commercial kit. **H**, **I** SLC7A11 and GPX4 mRNA expression and protein level in MAECs were detected. **J** Cell death rate was detected by trypan blue staining. Each experiment was repeated three times independently. Measurement data are depicted as mean ± SD. The *t* test was used for the comparisons between two groups. One-way ANOVA was employed for the comparisons among multiple groups, followed by Tukey’s multiple comparisons test. *p < 0.05.

Inhibition of ferroptosis can reduce lipid peroxidation and endothelial dysfunction of mouse aortic ECs, thereby alleviating AS [20]. To further explore whether EV-miR-199a-3p reduced AS by inhibiting ferroptosis of ECs, we used lentivirus to intervene miR-199a-3p expression in EPCs. miR-199a-3p expression in the anti-miR-199a-3p group was lower than that in the control group (Fig. 3A). Compared with that in the anti-miR-NC group, miR-199a-3p expression in the anti-miR-199a-3p group was significantly reduced (Fig. 3B).

Then, EVs/EVs-anti miR-199a-3p were co-cultured with ox-LDL-treated MAECs, and the results showed that compared with that in the ox-LDL group, miR-199a-3p expression in the EVs-anti miR-NC group was significantly elevated; compared with that in the EVs-anti miR-NC group, miR-199a-3p expression in the EVs-anti miR-199a-3p group was significantly reduced (Fig. 3C). Compared with that of the control group, the cell viability of the ox-LDL group was decreased; compared with that of the ox-LDL group, the cell viability of the EVs-anti miR-NC group was increased; and compared with that of the EVs-anti miR-NC group, the cell viability of the EVs-anti miR-199a-3p group was decreased (Fig. 3D). Lactate dehydrogenase (LDH) content in the supernatant was detected to measure the cell injury after ox-LDL treatment. Compared with that of the control group, LDH level of the ox-LDL group was significantly elevated, while LDH level was decreased after EVs introduction; compared with that of the EVs-antimiR-NC group, LDH level of the EVs-anti miR-199a-3p group was notably increased (Fig. 3E). Glutathione (GSH) consumption, lipid reactive oxygen species (ROS) production, lipid peroxidation, and iron accumulation are important indicators of the activation of ferroptosis signaling pathway. Compared with the control group, the ox-LDL group had lower GSH consumption, higher lipid ROS production, lipid peroxidation, and iron accumulation, and lower expressions of ferroptosis-related factors (SLC7A11 and GPX4). The introduction of EVs increased GSH consumption, decreased lipid ROS production, lipid peroxidation, and iron accumulation, and increased the expressions of ferroptosis-related factors (SLC7A11 and GPX4). Compared with the EVs-anti miR-NC group, the EVs-anti miR-199a-3p had increased lipid ROS production, lipid peroxidation, and iron accumulation, and decreased GSH consumption and expressions of ferroptosis-related factors (SLC7A11 and GPX4) (Fig. 3F–I). Trypan blue staining showed that cell death rate was increased in the ox-LDL group compared with that in the control group, but decreased in the EVs-anti miR-NC group compared with that in the ox-LDL group, while cell death rate was increased in the EVs-anti miR-199a-3p group compared with that in the EVs-anti miR-NC group (Fig. 3J). Briefly, EPC-EVs inhibited ferroptosis of ECs through miR-199a-3p.

### miR-199a-3p targeted SP1

**Fig. 4:**
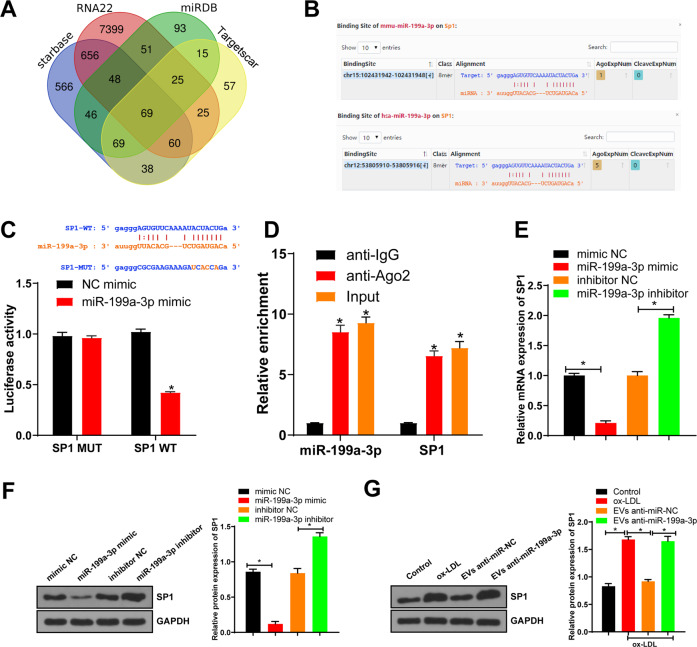
miR-199a-3p targeted SP1. **A** The target genes of miR-199a-3p were predicted through TargetScan and Starbase databases. **B** The binding site between miR-199a-3p and SP1 was predicted through the Starbase database. **C** The binding relationship between miR-199a-3p and SP1 was verified using dual-luciferase assay. **D** RNA immunoprecipitation of Ago2 antibody by MAEC lysate. **E** miR-199a-3p expression in each group was detected using qRT-PCR. **F** SP1 expression in each group was detected using western blot. **G** SP1 expression in MAECs after EPC-EVs treatment was detected using western blot. Each experiment was repeated three times independently. Measurement data are depicted as mean ± SD. The *t* test was used for the comparisons between two groups. One-way ANOVA was employed for the comparisons among multiple groups, followed by Tukey’s multiple comparisons test. *p < 0.05.

To further study the molecular mechanism of miR-199a-3p regulating ferroptosis of ECs, we predicted the downstream target genes of miR-199a-3p through TargetScan and Starbase databases (Fig. 4A) and found that SP1 had a target binding site with miR-199a-3p in both mice and humans (Fig. 4B). Inhibition of SP1 represses ferroptosis [21, 22], and silencing SP1 inhibits endothelial cell injury [23]. Upregulation of SP1 promotes the progression of AS [24]. Therefore, we speculated that miR-199a-3p inhibited the progression of AS by targeting SP1 and then affected the ferroptosis of ECs. The fluorescence intensity of the miR-199a-3p mimic group was lower than that of the NC mimic group (Fig. 4C). Moreover, anti-Ago2 was utilized to carry out RNA immunoprecipitation (RIP) assay on MAEC extracts. As expected, miR-199a-3p and SP1 were enriched in the Ago2 pellets compared with the IgG control (Fig. 4D).

We further intervened miR-199a-3p expression in MAECs. SP1 expression in the miR-199a-3p mimic group was lower than that in the NC mimic group; compared with that in the NC inhibitor group, SP1 expression in the miR-199a-3p inhibitor group was increased (Fig. 4E, F). EPC-EVs were co-cultured with MAECs. Compared with that in the control group, SP1 expression in the ox-LDL group was increased; compared with that in the ox-LDL group, SP1 expression in the EVs-anti miR-NC group was decreased; and compared with that in the EVs-anti miR-NC group, SP1 expression in the EVs-anti miR-199a-3p group was increased (Fig. 4G). These results suggested that EPC-EVs delivered miR-199a-3p to inhibit SP1 in MAECs.

### EV-miR-199a-3p targeted SP1 and thereby inhibited ferroptosis of ECs

**Fig. 5:**
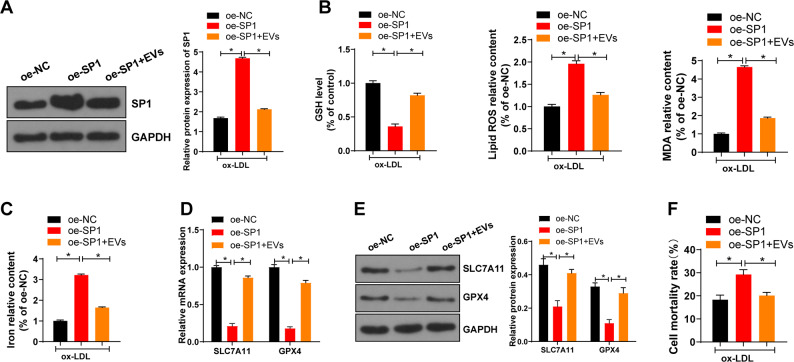
EV-miR-199a-3p targeted SP1 and thereby inhibited ferroptosis of ECs. **A** SP1 expression in each group was detected using western blot. **B** GSH consumption, lipid ROS production, and lipid peroxidation were detected using the kits. **C** The relative content of iron in MAECs was determined using the commercial kit. **D**, **E** SLC7A11 and GPX4 mRNA expression and protein levels in MAECs were detected. **F** Cell death rate was detected by trypan blue staining. Each experiment was repeated three times independently. Measurement data are depicted as mean ± SD. The *t* test was used for the comparisons between two groups. One-way ANOVA was employed for the comparisons among multiple groups, followed by Tukey’s multiple comparisons test. *p < 0.05.

To further investigate whether EV-miR-199a-3p affected the function of ECs by inhibiting SP1, we overexpressed SP1 in ox-LDL-treated MAECs and co-cultured them with EPC-EVs. Compared with that in the oe-NC group, SP1 expression in the oe-SP1 group was increased, while SP1 expression was decreased by further introducing EPC-EVs (Fig. 5A). The effect of SP1 on ferroptosis of ECs was further explored. Compared with the oe-NC group, the oe-SP1 group had reduced GSH consumption, increased lipid ROS production, lipid peroxidation, iron accumulation, and cell death rate, and decreased expressions of SLC7A11 and GPX4; compared with the oe-SP1 group, the oe-SP1 + EVs group showed increased GSH consumption, decreased lipid ROS production, lipid peroxidation, iron accumulation, and cell death rate, and elevated expressions of SLC7A11 and GPX4 (Fig. 5B–F). Briefly, miR-199a-3p targeted SP1 and thereby inhibited ferroptosis of ECs.

### EV-miR-199a-3p inhibited ferroptosis of ECs and thereby retarded AS

**Fig. 6:**
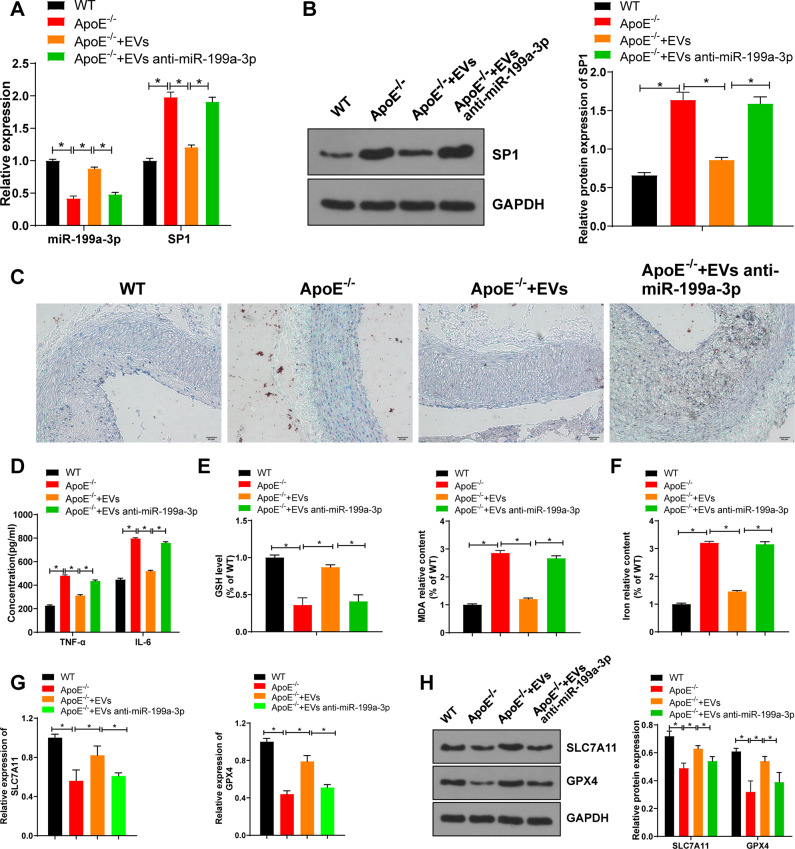
EVs miR-199a-3p inhibited ferroptosis of ECs and thereby retarded atherosclerosis. ApoE-/- mice were given a high-fat diet (HFD) for 4 weeks, and then the HFD-fed ApoE-/- mice were injected with EVs or not. **A** The expressions of miR-199a-3p and SP1 in the aorta of atherosclerotic mice were detected using qRT-PCR. **B** SP1 expression in the aorta of atherosclerotic mice was detected using western blot. **C** The area of aortic root plaque was observed by oil red O staining (scale bar = 20 μm). **D** The levels of inflammatory factors in serum of mice in each group were detected using ELISA. **E** GSH and MDA in the aorta of atherosclerotic mice were detected. **F** The iron content in aorta of atherosclerotic mice was detected. **G**, **H** SLC7A11 and GPX4 mRNA expression and protein levels in aorta of atherosclerotic mice were detected. *N* = 10. Measurement data are depicted as mean ± SD. The *t* test was used for the comparisons between two groups. One-way ANOVA was employed for the comparisons among multiple groups, followed by Tukey’s multiple comparisons test. *p < 0.05.

We further determined the role of EV-miR-199a-3p in the mouse model of AS. Compared with those in the WT mice, miR-199a-3p expression in the ApoE-/- mice was decreased, while SP1 mRNA expression and protein levels were increased significantly; compared with those in the ApoE-/- group, miR-199a-3p expression in the EVs group was increased, while SP1 mRNA expression and protein levels were decreased significantly; compared with those in the EVs group, miR-199a-3p expression in the EVs anti miR-199a-5p group was decreased, and SP1 mRNA expression and protein levels were increased (Fig. 6A, B). Compared with those in the WT group, the area of aortic plaque and serum TNF-α and IL-6 levels in the ApoE^-/-^ group were increased; compared with those in the ApoE^-/-^ group, the area of aortic plaque and serum TNF-α and IL-6 levels in the EVs group were decreased; compared with those in the EVs group, the area of aortic plaque and serum TNF-α and IL-6 levels in the EVs anti-miR-199a-5p group were increased (Fig. 6C, D).

Compared with the WT group, the ApoE^-/-^ group had decreased GSH consumption, increased lipid peroxidation and iron accumulation, and reduced expressions of SLC7A11 and GPX4; compared with the ApoE^-/-^ group, the EVs group had increased GSH consumption, decreased lipid peroxidation and iron accumulation, and elevated expressions of SLC7A11 and GPX4; and compared with the EVs group, the EVs anti-miR-199a-5p group had decreased GSH consumption, increased lipid peroxidation and iron accumulation, and reduced expressions of SLC7A11 and GPX4 (Fig. 6E–H). Altogether, EPC-EVs depressed EC ferroptosis through miR-199a-3p, thus delaying the occurrence of AS.

## Discussion

**Fig. 7:**
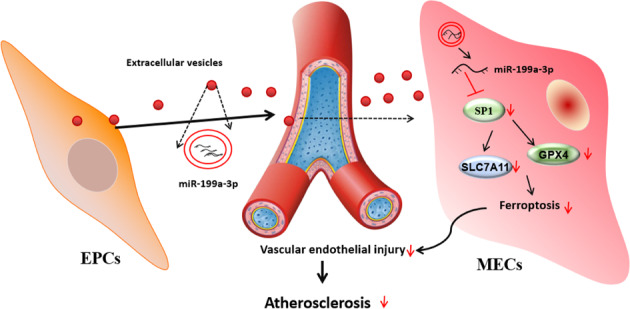
Mechanism diagram. EPCs secrete EVs to deliver miR-199a-3p and inhibit SP1, thereby inhibiting endothelial cell ferroptosis, delaying vascular endothelial injury, and ultimately alleviating atherosclerosis.

AS is a chronic cardiovascular disease and also one of the most frequent causes of death in the elderly [2]. Endothelial dysfunction is the starting point of AS and EPCs are closely associated with vascular endothelial function [25]. This study found that EPCs secreted EVs to deliver miR-199a-3p to inhibit SP1, thereby inhibiting EC ferroptosis and delaying vascular endothelial injury, and ultimately alleviating AS (Fig. 7).

Endothelial dysfunction in the lesion-prone areas of arterial vascular system is a critical factor in the pathology of atherosclerotic CVDs [26]. EPCs derived from bone marrow can differentiate into ECs, and once EC dysfunction, EPCs enter the blood circulation and replace the damaged cells [27]. As EPCs contribute to tissue vascularization after ischemic events, EPCs have great prospects in vascular repair and cardiovascular disease treatment [9]. EPC-derived EVs are demonstrated to facilitate EC angiogenesis by activating the Erk1/2 signaling pathway, which eventually promotes wound repair and regeneration [28]. Importantly, in the mouse model of atherosclerotic diabetes, EPC-EVs reduce the production of atherosclerotic plaques and the secretion of inflammatory factors, thus improving atherosclerotic endothelial dysfunction [13]. In the current study, EPCs were isolated and purified from mouse umbilical cord blood and EPC-EVs were further isolated from the conditioned medium of EPCs. We found that EPCs transferred EVs into ECs. It is well established that EVs enclose miRNAs to deliver genetic signaling into cells and in turn modulate cellular functions [29]. The next-generation sequencing shows that miR-199a-3p constitutes one of the top 10 miRNAs expressed in EPCs-derived EVs [13]. miR-199a-3p is related to vascular EC injury triggered by type 2 diabetes mellitus and miR-199a-3p may play a protective role in vascular endothelium [20]. Our results demonstrated that miR-199a-3p expression was decreased with the increase of plaque lesion area and serum pro-inflammatory factors; after ox-LDL treatment in vitro, miR-199a-3p expression in ECs was significantly reduced. miR-199a-3p exerts favorable effects on asymptomatic AS by repressing vascular smooth muscle cell proliferation and migration [30]. These results revealed that miR-199a-3p expression was associated with AS lesion. Hence, we then further investigated whether EPC-EVs affected AS by transferring miR-199a-3p and our results confirmed that EPC-EVs transferred miR-199a-3p into ECs.

Ferroptosis is regulated by iron-dependent lipid peroxidation and associated with diverse pathophysiological processes and diseases including CVDs [31]. Accordingly, we hypothesized that EVs carrying miR-199a-3p attenuated AS by inhibiting ferroptosis of ECs. Therefore, we used lentivirus to intervene miR-199a-3p expression in EPCs, and then extracted EVs (EVs-anti miR-199a-3p). Thereafter, EVs-anti miR-199a-3p was co-cultured with ox-LDL-treated MAECs. We found that cell viability was increased after treatment with EVs, but was decreased after treatment with EVs-anti miR-199a-3p. Meanwhile, cell death rate was decreased after treated with EVs, but was increased after treated with EVs-anti miR-199a-3p. Ferroptosis relies on ROS generation and iron accumulation. Excessive lipid peroxidation causes ferroptosis and the repletion and depletion of intracellular iron and GSH aggravate this phenotype respectively [32]. GPX4 is an antioxidant enzyme for neutralizing lipid peroxides, and inactivation of GPX4 causes lipid ROS accumulation and eventually results in ferroptosis [33]. SLC7A11 is a component of cystine/glutamate antiporter system x_c_^-^ and suppression of SLC7A11 reduces GSH production and inactivates GPX4 [34]. We found that EVs increased GSH consumption, decreased lipid ROS production, lipid peroxidation and iron accumulation, and increased the expressions of SLC7A11 and GPX4, while the introduction of EVs-anti miR-199a-3p showed the opposite trend. In addition, the in vivo results were consistent with the above results. Inhibition of ferroptosis can reduce lipid peroxidation of aortic ECs in mice and endothelial dysfunction, and thus alleviate AS [6]. EPC-EVs alleviate the symptoms of steroid-induced osteoporosis by suppressing the activation of the ferroptotic pathway [14]. These results suggested that EVs inhibited the ferroptosis of ECs and delayed the occurrence of AS by carrying miR-199a-3p.

Subsequently, we explored the molecular mechanism of miR-199a-3p regulating ferroptosis of ECs. The target genes of miR-199a-3p were predicted through databases and we found that SP1 binds to miR-199a-3p in both mice and human. SP-1 is a widely expressed positive transcription factor, and it regulates inflammatory repair process and represents an important target for the treatment of atherosclerotic vascular diseases [23, 35]. The SP-1/miR-135b/HIF-1α axis modulates vascular endothelial injury induced by hypoxia [23]. Our relevant experiments confirmed that EPC-EVs could target SP1 in MAECs by delivering miR-199a-3p. Inhibition of the ferroptotic pathway STAT4/SP1 signaling can regulate iron metabolism, thus protecting neurons from ferroptosis [22]. Our functional rescue experiment showed that overexpression of SP1 could reverse the protective effect of EPC-EVs on ferroptosis of ECs. Upregulating SP1 is also demonstrated to promote the progression of AS [24]. SP1 increases the transcription of ACSl4, which aggravates ferroptosis and intestinal ischemia-reperfusion injury [21]. Briefly, EPC-EVs carrying miR-199a-3p affected the function of ECs and inhibited ferroptosis of ECs by targeting SP1.

To sum up, EPC-EVs inhibited ferroptosis of ECs via the miR-199a-3p/SP1 axis and ultimately alleviated AS. This pilot study may provide theoretical holds for the EPC-EV-based therapeutic regimens of AS patients. In the future, we will conduct more prospective trials on the feasibility and safety of EPC-EVs in the treatment of AS, so as to refine our clinical guidance.

## Materials and methods

### Ethics statement

This study was performed following the approval of the Ethical Committee of Qingdao Hiser Medical Center. All the experiments were implemented on the guide for the care and use of laboratory animals.

### Cell isolation and culture

To isolate EPCs, we used anti-CD133 coupled magnetic beads (Miltenyi Biotech, Bergisch Gladbach, Germany) to screen the cells expressing early EPC surface marker CD133 from the cord blood of C57BL/6 mice. Then, EPCs were cultured in Dulbecco’s modified Eagle’s medium (DMEM) containing 10% fetal bovine serum (FBS). The cell morphology was observed using an inverted microscope.

By referring to the modified method of Kobayashi et al., mouse aortic endothelial cells (MAECs) were isolated from the aorta of C57BL/6 mice. Briefly, the mice were dissected from the midline of the abdomen and the chest was opened. The aorta of mice was isolated and washed with phosphate-buffered saline (PBS). The aorta was cut longitudinally to remove the fat or connective tissues, followed by washing with serum-free DMEM (12100-46, GIBCO, Grand Island, NY, USA). Next, the aorta was incubated with collagenase II solution (171010115, Thermo Fisher Scientific, Waltham, MA, USA) at 37 °C for 45 min. Thereafter, the aorta was isolated from the adventitia and rinsed with DMEM (F8067, Sigma-Aldrich, Merck KGaA, Darmstadt, Germany) containing 20% FBS to collect MAECs. Next, the cells were centrifuged and cultured in DMEM containing 20% FBS at 37 °C for 2 h. Afterward, cells were rinsed with PBS and cultured in the fresh medium. After 1 week, the cells were passaged upon reaching 90% confluence. MAECs at passage 3 were used for subsequent experiments.

### Immunofluorescence

EPCs were fixed with 40 g/L polyformaldehyde for 15 min, rinsed with PBS three times, blocked with 10% goat serum, and incubated with the primary antibodies CD34 (ab110643, Abcam Inc., Cambridge, MA, USA), CD133 (ab222782, Abcam), vWF (ab6994, Abcam), and VEGF-2 (ab2349, Abcam) at 4 °C overnight. Then, the cells were added with TRITC-labeled fluorescent secondary antibody goat anti-rabbit IgG H&L (ab6718, 1:1000, Abcam) and FITC-labeled fluorescent secondary antibody goat anti-mouse IgG H&L (ab6785, 1:1000, Abcam) for 45 min. Following two PBS washings, the cells were cultured with Hoechst3342 for 15 min, washed, and finally photographed using a fluorescent microscope.

### Cell transfection and lentivirus infection

EPCs were infected with miR-199a-3p lentivirus silencing vector (anti-miR-199a-3p) and its negative control (anti-miR NC). MAECs were infected with specificity protein 1 (SP1) lentivirus overexpression vector (oe-SP1) and its negative control oe-NC, and transfected with NC mimic, miR-199a-3p mimic, NC inhibitor, or miR-199a-3p inhibitor.

For lentivirus infection, GFP-labeled miR-199a-3p lentiviral silencing vector and SP1 lentiviral overexpression vector (both purchased from Genechem, Shanghai, Shanghai) were transfected into 293T cells (CRL-1573, American Type Culture Collection, Manassas, VA, USA). From 24 h to 72 h after transfection, the supernatant containing lentivirus particles was collected every 12 h and filtered through a 0.45 μm cellulose acetate filter. The final virus titer was 2 × 10^10^ TU/mL. One day before lentivirus infection, EPCs and MAECs were seeded into the six-well plates (1 × 10^5^ cells/mL). When the cell confluence reached 50–70%, the culture medium was sucked out. Each well was added with 2 × 10^4^ TU complete culture medium containing 50-fold dilutions of the virus and 10 μg/mL Polybrene (H8761, Solarbio, Beijing, China) and cultured at 37 °C with 5% CO_2_. After 48–72 h of transfection, the cells stably infected with lentivirus were screened in the medium containing 0.5 μg/mL puromycin (A1113803, Invitrogen Inc., Carlsbad, CA, USA). The medium was refreshed every 3 days and the cell line with stable expression was obtained.

mimic NC (50 nM), miR-199a-3p mimic (50 nM), inhibitor NC (150 nM), and miR-199a-3p inhibitor (150 nM) were purchased from Ribobio (Guangzhou, China). ECs were seeded into the 24-well plates (5 × 10^5^ cells/well) and transfected using Lipofectamine 3000 (Invitrogen). The final volume of each well was 500 μL.

### Isolation and purification of EPC-EVs

The culture medium/serum was ultracentrifuged at 100,000 × *g* and 4 °C overnight to remove the EVs from the serum. EPCs were cultured in endothelial basal medium (EBM-2; Longza, Allendale, NJ, USA) supplemented with EBM-2 SingleQuots, 10% EV-free FBS (System Biosciences, Palo Alto, CA, USA), and 1% penicillin and streptomycin (GIBCO, Gaithersburg, MD, USA) for 48 h. The culture medium was collected and centrifuged at 500 × *g* and 4 °C for 15 min to eliminate cell debris, centrifuged at 2000 × *g* and 4 °C for 15 min to remove cell debris or apoptotic bodies, following centrifugation at 10,000 × *g* and 4 °C for 20 min to remove large vesicles. The cells were filtered with a 0.22 μM filter, centrifuged at 110,000 × *g* and 4 °C for 70 min, resuspended with PBS, and then ultracentrifuged under the same conditions, and finally resuspended with 100 μL sterile PBS.

### Identification of EVs

For Nanosight nanoparticle tracking analysis (NTA), 20 μg EVs were dissolved in 1 mL PBS for 1 min to retain the uniform distribution of EVs. Then, EV particle size distribution was directly measured using a Nanosight nanoparticle tracking analyzer (Malvern Panalytical, UK).

For transmission electron microscopy (TEM), 20 μL ultracentrifuged EVs were loaded to carbon-coated copper electron microscope grids for 2 min, followed by negatively 5-min of staining with phosphotungstic acid solution (12501-23-4, Sigma-Aldrich). The grid was then washed with PBS three times to eliminate excess dye solution and kept semi-dry with filter paper. The images were observed using a TEM (H7650, Hitachi, Tokyo, Japan) at 80 kV.

The surface markers of EVs were identified using western blot. EV suspension was concentrated, and then, total protein concentration was examined using a bicinchoninic acid (BCA) assay kit (23227, Thermo Fisher Scientific). SDS-PAGE gel was prepared and protein denaturation and electrophoresis were performed. Afterward, the expressions of EV specific markers CD9 (ab92726, Abcam), CD81 (ab92726, Abcam), Alix (ab76608, Abcam), and calnexin (ab22595, Abcam) were detected.

### Package of miR-199a-3p in EVs

RNase A treatment was used to determine whether miR-199a-3p was surface bound to or packaged in EVs. EVs were resuspended in PBS and then incubated with 2 μg/μL RNase A at 37 °C for 20 min. EV membrane integrity was destroyed using the detergent. After 20 min of 0.1% Triton X-100 treatment, the above RNase A treatment was carried out. After RNase A incubation, the lysis buffer was added to inhibit the reaction, and RNA was isolated.

### Uptake of EVs

Purified EPC-EVs were labeled using a PKH67 green fluorescent kit (Sigma-Aldrich). EVs were resuspended in 1 mL Diluent C, and 4 × 10^−6^ M dye solution was prepared by adding 4 μL PKH67 ethanol dye solution to 1 mL Diluent C. Next, 1 mL EV suspension was stained with dye solution for 5 min and cultured with 2 mL 1% EV-free FBS for 1 min to terminate dyeing. EVs were enriched in the sucrose (1.13–1.19 g/mL) by centrifuging at 100,000 × *g* for 2 h [36]. PKH67-labeled EVs were treated with ECs for 12 h-incubation at 37 °C. The cells were fixed with 4% paraformaldehyde and washed with PBS, and the nucleus was stained with 4′,6-diamidino-2-phenylindole (DAPI) (Sigma-Aldrich). As for the detection of the uptake of EPC-EVs carrying Cy3-miR-199a-3p by ECs, EPCs were transfected with Cy3-miR-199a-3p (GenePharma, Shanghai, China). After 6 h, the culture medium was changed to 10% EV-free serum medium for 48-h incubation. The supernatant was collected, centrifuged, resuspended with PBS, and added to ECs. Similarly, the cells were fixed with 4% paraformaldehyde, washed with PBS, and the cytoskeleton was labeled with Phalloidin-iFluor 488 Reagent (1:1000, ab176753, Abcam) at room temperature for 30 min. The nucleus was stained with DAPI. Finally, the uptake of EVs by ECs was observed under the confocal microscope (LSM710, Zeiss, Germany).

### Oxidized low-density lipoprotein treatment

Oxidized low-density lipoprotein (ox-LDL) can induce cell death. MAECs were cultured in DMEM containing 20% FBS at 37c with 5% CO_2_. The cells were assigned into control group, ox-LDL group, EVs anti-NC group, EVs miR-199a-3p group, oe-NC group, oe-SP1 group, and oe-SP1 + EVs group. Briefly, the MAECs in the ox-LDL group were treated with 100 μg/mL ox-LDL, or co-treated with 100 μg/mL ox-LDL and 4 μL EVs (1 × 10^9^ particles/mL) for 24 h.

### MTT assay

Cell viability was assessed using MTT assay. Briefly, cells were seeded to the 96-well plates (2 × 10^3^ cells/well) containing 100 μL complete medium. After co-incubation, each well was added with 20 μL MTT solution (5 mg/mL) at 37a for 4 h. Then, each well was added with 150 μL dimethyl sulphoxide and cultured at 37n for 20 min. The optical density (OD) value was read at a wavelength of 490 nm, and each group had three duplicated wells.

### Detection of lactate dehydrogenase

The supernatant of cell culture was collected, and the activity of LDH was detected using the LDH assay kit (ab102526, Abcam). Briefly, 25 μL cell supernatant was mixed with 25 μL substrate and cultured at 37 °C for 15 min. Then, the sample was added with 2,4-dinitrophenylhydrazine at 37 °C for 15 min and cultured with 250 μL 0.4 M NaOH for 5 min. The absorbance was measured on the Evolution^TM^ spectrophotometer (840-210600, Thermo Fisher Scientific).

### Trypan blue staining

Cells in the logarithmic growth phase with good growth conditions were obtained and centrifuged for 10 min at 1200 × *g* to remove the supernatant. Cells were added with an appropriate amount of culture medium and resuspended to obtain single cell suspension cultured in serum-free conditions. Cell concentration was calculated and adjusted to 4.0 × 10^6^ cells/bottle. Cells were treated with EVs for 1 h at 37 °C. Subsequently, 900 μL cell fluid was collected after thorough mixture and added with 100 μL 0.4% trypan blue and mixed thoroughly, followed by observation under the microscope after 1-min staining. The blood cell counting plate was covered with the cover slip. One drop of stained cell suspension was dripped onto the top and bottom edges of the counting plate with a dropper to fill the gap between the blood cell counting plate and cover slip. Dead cells were observed and counted under the microscope and cell death rate was calculated. The experiment was repeated three times independently to obtain average value. Cell death rate (%) = dead cell number/total cell number × 100%.

### Detection of glutathione and malondialdehyde

GSH (A006-2) and malondialdehyde (MDA) (A003-1) levels in the thoracic aorta tissue and MAECs were measured according to the manufacturer’s instructions of commercial kits. All the kits are purchased from Nanjing Jiancheng Bioengineering Research Institute (Nanjing, Jiangsu, China).

### Iron determination

Total iron in cell lysate and tissue supernatant was quantified using an iron detection kit (ab83366, Abcam). In short, the particles of isolated tissues and cultured MAECs were homogenized with saline and PBS and centrifuged at 16,000 × g for 10 min to remove the insoluble matters. Then, 5 µL iron reducing agent was added to 50 µL sample to get the total iron content (Fe ^3+^and Fe ^2+^). Next, 100 µL iron probe solution was added to the sample and incubated at 25 °C in the dark for 60 min. The absorbance at 593 nm was measured using a spectrophotometer. Each experiment was repeated three times.

### Detection of reactive oxygen species

The lipophilic fluorescent dye C11-BODIPY581/591 (D3861, Journal Pre-proof Gibco) was used to determine the lipid ROS level in MAECs. Briefly, after treatment and incubation, the cells were collected and washed with PBS. The lipid ROS was labeled with 5 μmol/L C11-BODIPY581/591 at 37 °C for 30 min. Following PBS washing three times, the cells were resuspended in 500 mL PBS. The fluorescence activity was analyzed using a flow cytometer (NovoCyte, Aceabio, San Diego, CA, USA). With unstained cells as the negative control and 50 μg/mL oxidant rosup-cultured (S0033S, Beyotime, Shanghai, China) cells as the positive control, ROS was detected by fluorescence.

### Quantitative real-time polymerase chain reaction

The total RNA was extracted using TRIzol reagent (Invitrogen) and reverse transcribed into cDNA using Prime Script RT kit (Takara, Dalian, China). miRNA cDNA was synthesized from total RNA of cells and tissues using miRcute Plus miRNA First Strand cDNA synthesis kit (TianGen Biotech Co., Ltd. Beijing, China). The synthesized exogenous reference cel-miR-39 (1 pmol per sample; TianGen) was added to 350 µL culture medium or 100 µg EVs in advance. Real-time PCR of mRNA was performed using the SYBR Premix Ex Taq kit (Takara) and ABI StepOne real-time PCR system (Applied Biosystems, Inc., Carlsbad, CA, USA), with GAPDH as the internal reference. Real-time PCR of miRNA was conducted using miRcute Plus miRNA qPCR kit (TianGen), with U6 as the internal reference. In addition, the miRNA level in the medium and EVs were standardized according to the exogenous internal reference cel-miR-39. The relative transcription level of target genes was calculated using the 2-^△△CT^ method. △△Ct = △Ct experimental group-△Ct control group, in which △Ct = Ct target gene-Ct internal reference gene. Ct is the number of amplification cycles when the real-time fluorescence intensity of the reaction reaches the set threshold. At this time, the amplification is logarithmic. Each experiment was repeated three times. The primer sequences are presented in Table 1.

**Table 1:** Primer sequence for RT-qPCR.

Gene	Primer sequence (5’-3’)	
GPX4	F: CAACCAGTTTGGGAGGC	R: CTTGGGCTGGACTTTCAT
SLC7A11	F: TTGGAGCCCTGTCCTATGC	R: CGAGCAGTTCCACCCAGAC
SP1	F: CTACCCCTACCTCAAAGG	R: CTCTCCTTCTTTTTGCTGG
GAPDH	F: GGTGAAGGTCGGTGTGAACG	R: CTCGCTCCTGGAAGATGGTG
miR-199a-3p	F: ACACTCCAGCTGGGACAGTAGTCTGCACAT	R: TGGTGTCGTGGAGTCG
U6	F: CTCGCTTCGGCAGCACA	R: TGGTGTCGTGGAGTCG
cel- miR-39	F: GGTCACCGGGTGTAAATCAGCTTG.	R: TGGTGTCGTGGAGTCG

### Western blot

The protein was lysed in lysis solution supplemented with phosphatase inhibitor, protease inhibitor, and phenylmethylsulfonyl fluoride, followed by the measurement of protein concentration using a BCA kit (Thermo Fisher Scientific). Then, 10–20 µg protein was sampled on 8–12% 30% acrylamide-Bis gel and then transferred onto 0.22 µm PVDF membranes. Subsequently, the membranes were treated with 5% skim milk for 1 h and incubated with antibodies GAPDH (ab8245, Abcam), SP1 (ab227383, Abcam), GPX4 (ab125066, Abcam), and SLC7A11 (ab175186, Abcam) overnight. All antibodies were diluted according to the instructions. Next day, the membranes were cultured with the secondary antibody peroxidase-conjugated goat anti-rabbit IgG (H&L) (#111035003, Jackson ImmunoResearch, USA) for 1 h and developed with luminescent liquid (Thermo Fisher Scientific). Image J was used for analysis. The relative protein content was expressed as the gray value of the corresponding protein band/the gray value of GAPDH protein band. The experiment was repeated three times.

### Dual-luciferase reporter assay

The synthetic vector Vector-SP1 3′UTR gene fragment was introduced into pmiRGLO dual-luciferase miRNA target expression vector (E1330, Promega, Madison, WI, USA). The mutation site of binding site was designed on the wild-type SP1. The target segment was inserted into pmiRGLO vector by T4 DNA ligase after restriction endonuclease. The above operations were completed by Genechem (Shanghai, China). The luciferase reporter plasmids were co-transfected with miR-199a-3p mimic into HEK293T cells. The cells were lysed after 48 h. The dual-luciferase reporter assay system kit (E1910, Promega) and Luminometer TD-20/20 instrument (E5311, Promega) were utilized to measure luciferase activity. The experiment was repeated three times.

### RNA immunoprecipitation

RIP was performed using the Magna RIP kit (Millipore). In short, MAECs were lysed in radio-immunoprecipitation assay (RIPA) lysis buffer and centrifuged at 12,000 × *g* and 4 °C for 10 min to obtain the supernatant. One part of the cell extract was taken out as input, and the other part was incubated with antibody for co-precipitation. The specific steps were as follows: 50% μL magnetic beads in each co-precipitation reaction system were washed and suspended in 100 μL RIP wash buffer, and 5 μg antibody was added according to the experimental grouping for binding. The magnetic bead-antibody complex was washed and resuspended in 900 μL RIP wash buffer, supplemented with 100 μL cell extracts, and cultured at 4 °C overnight. The sample was placed on the magnetic stand for the collection of magnetic bead-protein complex. The sample and input were detached by proteinase K and then RNA was extracted for subsequent PCR detection. The antibody AGO2 (1:50, ab32381, Abcam) was mixed at room temperature for 30 min, and IgG (1:100, ab109489, Abcam) was used as NC.

### Animal experiment

Male C57BL/6 mice and ApoE^-/-^ mice aged 6–8 weeks were purchased from Shanghai SLAC Laboratory Animal Co., Ltd (Shanghai, China) and raised in a specific pathogen-free grade animal room at 20–22 °C with 40–60% humility and maintained in a 12 h light/dark cycle, with free food and water. The mice used in the experiment were kept in the animal room for at least 1 week.

Thirty ApoE^-/-^ mice were fed an atherosclerotic diet (#D12108c, Research Diets Inc., New Brunswick, NJ, USA) containing high cholesterol (1.25%) to induce AS. After 4 weeks of feeding, 100 μg/kg EVs (EVs and EVs anti-miR 199a-3p) was injected into each mouse via tail vein every day for 3 days. After injection, the mice were raised in the animal experimental center. The remaining mice were injected with the same dose of PBS. The wild-type C57BL/6 mice served as normal controls (*N* = 10). After 2 weeks, the mice were anesthetized by pentobarbital sodium and then killed. The heart blood was collected by the cardiac puncture method. After PBS perfusion, the thoracic aorta was collected and fixed in 4% paraformaldehyde for 24 h, then placed in 30% sucrose-PBS buffer, embedded and frozen at the optimal cutting temperature.

### Oil red O staining

Briefly, 10 μm fresh frozen sections were rinsed with 78% methanol for 1 min, and then 60 mL ORO working solution (Muto Pure Chemicals Co., Ltd., Bunkyo-ku, Japan) was added into distilled water to prepare a double dilution. After mixing and standing for 10 min, the sections were incubated with the diluent for 50 min. After that, the sections were incubated in 78% methanol, Meyer heme, and 0.05% lithium carbonate each for 1 min (the sections were washed with tap water after each step). The sections were observed and photographed after drying. The following formula was used to calculate the lesion area (%): ORO-positive staining area/total lesion area × 100%.

### Enzyme-linked immunosorbent assay

Briefly, 3 mL heart blood of the mice was centrifuged for 15 min to collect the upper serum. The serum samples were kept in the dry sterile EP tube at −80 °C. The frozen serum samples were taken out and naturally dissolved at room temperature. The levels of TNF-α and IL-6 in mouse serum were detected using the ELISA kits. The absorbance (A) of each well at 450 nm was measured using the microplate reader (Synergy 2, BioTek). The regression equation of the standard curve was calculated with the standard concentration as abscissa and A value as ordinate. The A value of sample was substituted into the equation to calculate the concentration of target protein in the sample.

### Statistical analysis

Statistical analyses were introduced using SPSS 21.0 (IBM Corp. Armonk, NY, USA) and GraphPad Prism 6.0 (GraphPad Software, San Diego, CA, USA). Measurement data are depicted as mean ± standard deviation. The unpaired *t* test was employed for the comparisons between two groups. One-way analysis of variance (ANOVA) was employed for the comparisons among multiple groups, followed by Tukey’s multiple comparisons test. A value of *p* < 0.05 was regarded statistically significant.

## Supplementary information

WB 1E

WB 3I

WB 4F

WB 4G

WB 5A

WB 5E

WB 6B

WB 6H

Attribution of Authorship

## Data Availability

All the data generated or analyzed during this study are included in this published article.
