# Supplementary figures and images for: Effect of endothelial progenitor cell-derived extracellular vesicles on endothelial cell ferroptosis and atherosclerotic vascular endothelial injury
Source: Cell Death Discov. 2021 Sep 7;7:235. doi: 10.1038/s41420-021-00610-0 (PMC8423825; doi:10.1038/s41420-021-00610-0)

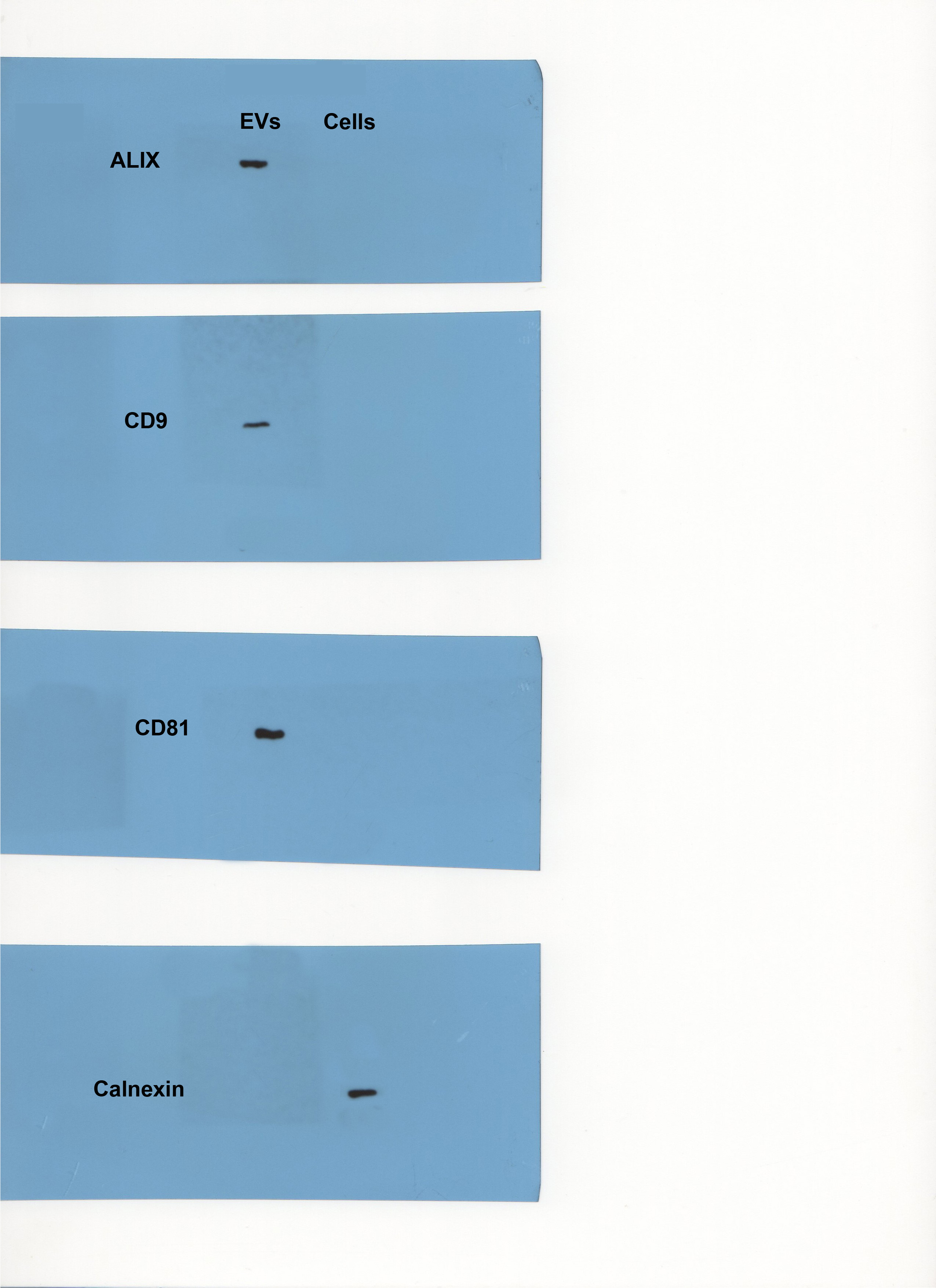

Supplement: Supplementary file 1 — WB 1E [file 41420_2021_610_MOESM1_ESM.jpg]

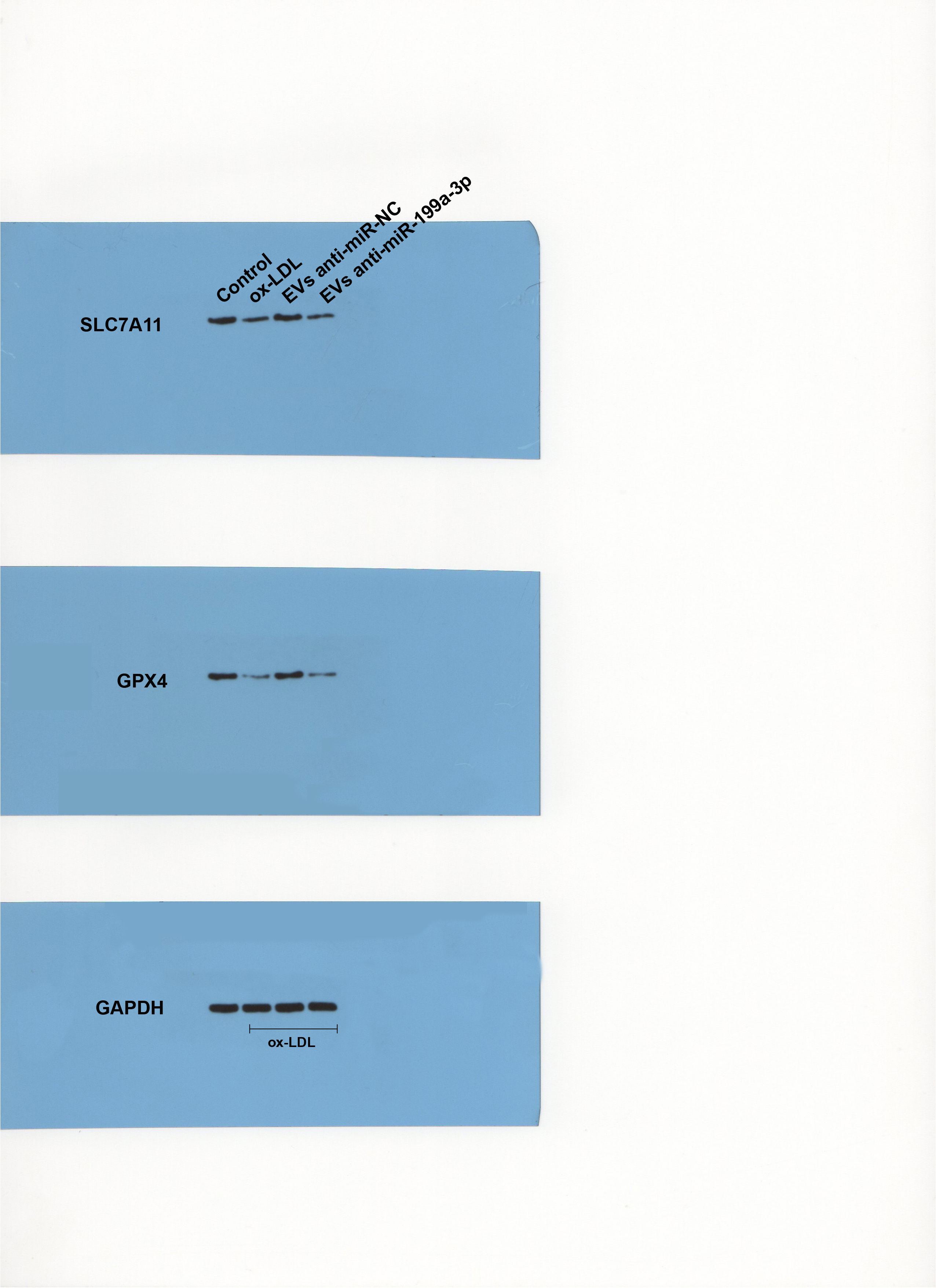

Supplement: Supplementary file 2 — WB 3I [file 41420_2021_610_MOESM2_ESM.jpg]

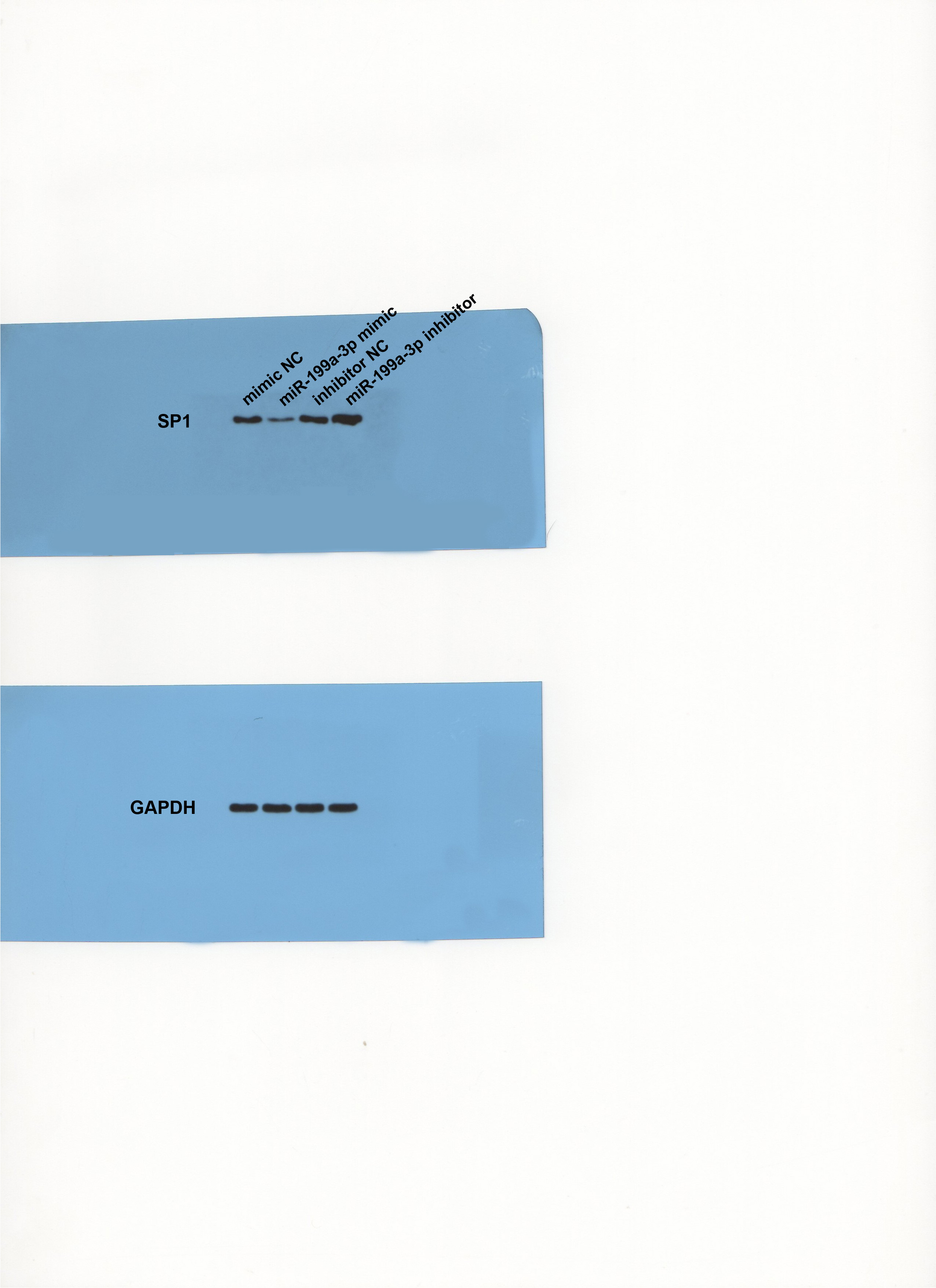

Supplement: Supplementary file 3 — WB 4F [file 41420_2021_610_MOESM3_ESM.jpg]

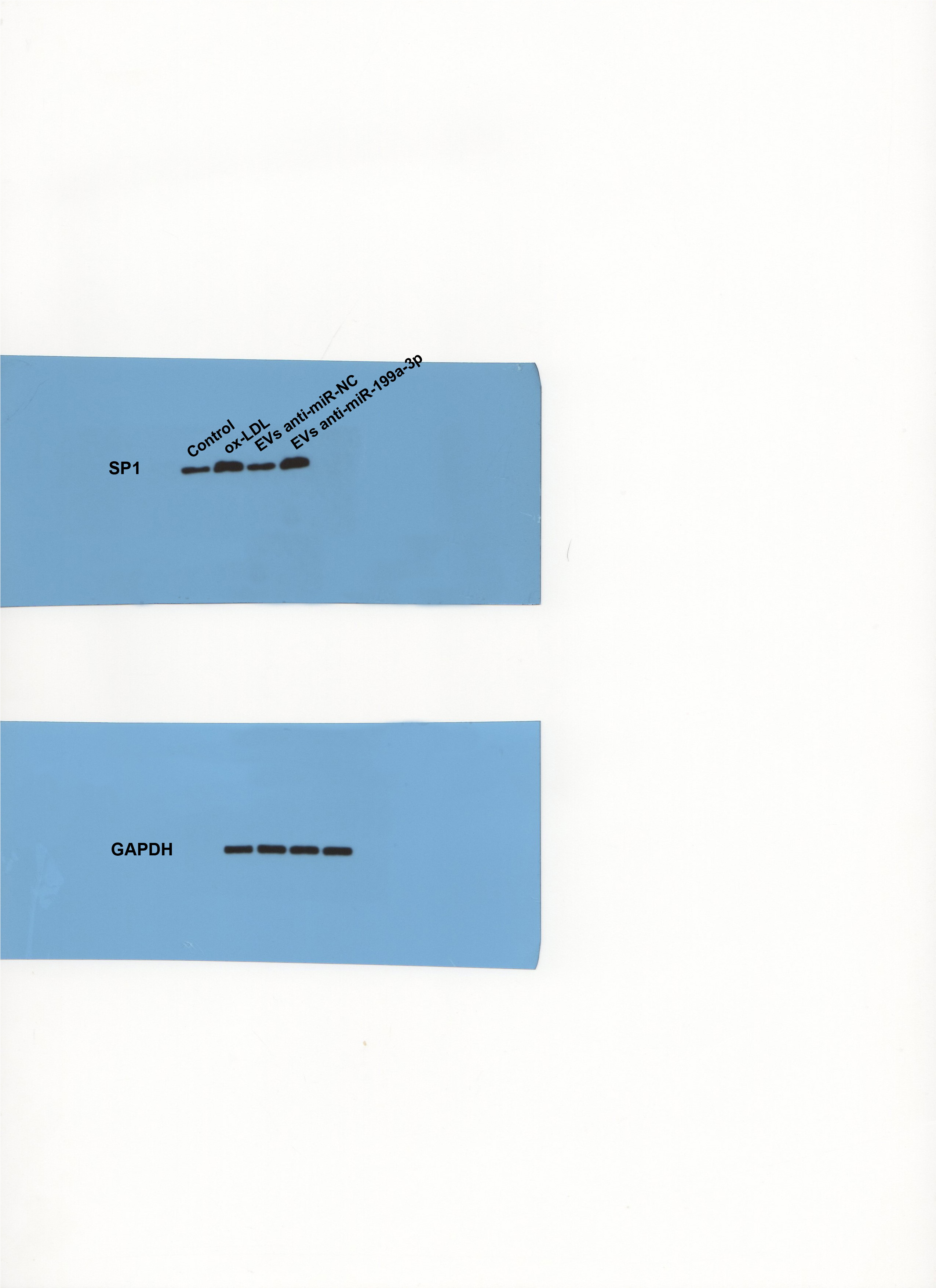

Supplement: Supplementary file 4 — WB 4G [file 41420_2021_610_MOESM4_ESM.jpg]

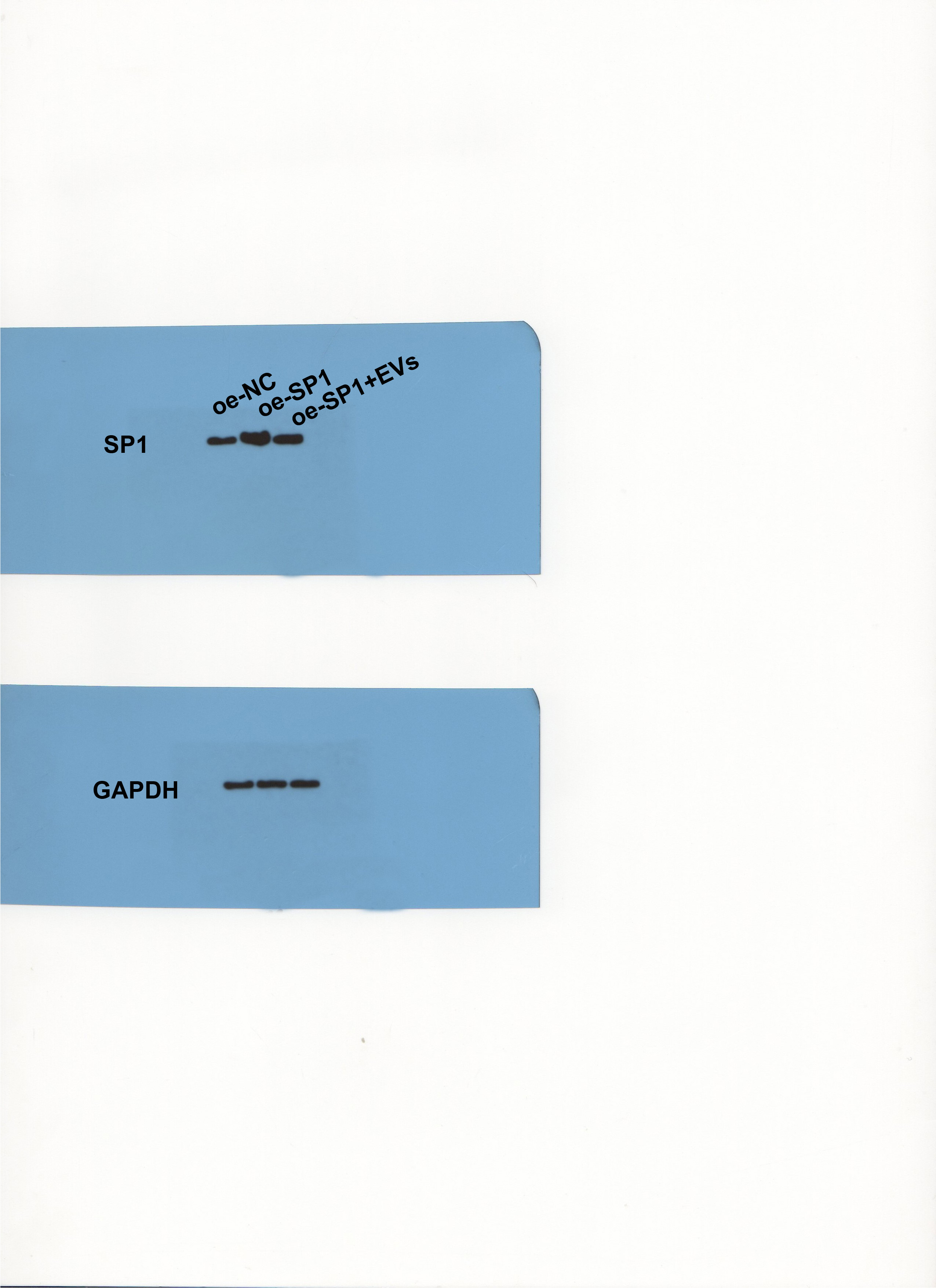

Supplement: Supplementary file 5 — WB 5A [file 41420_2021_610_MOESM5_ESM.jpg]

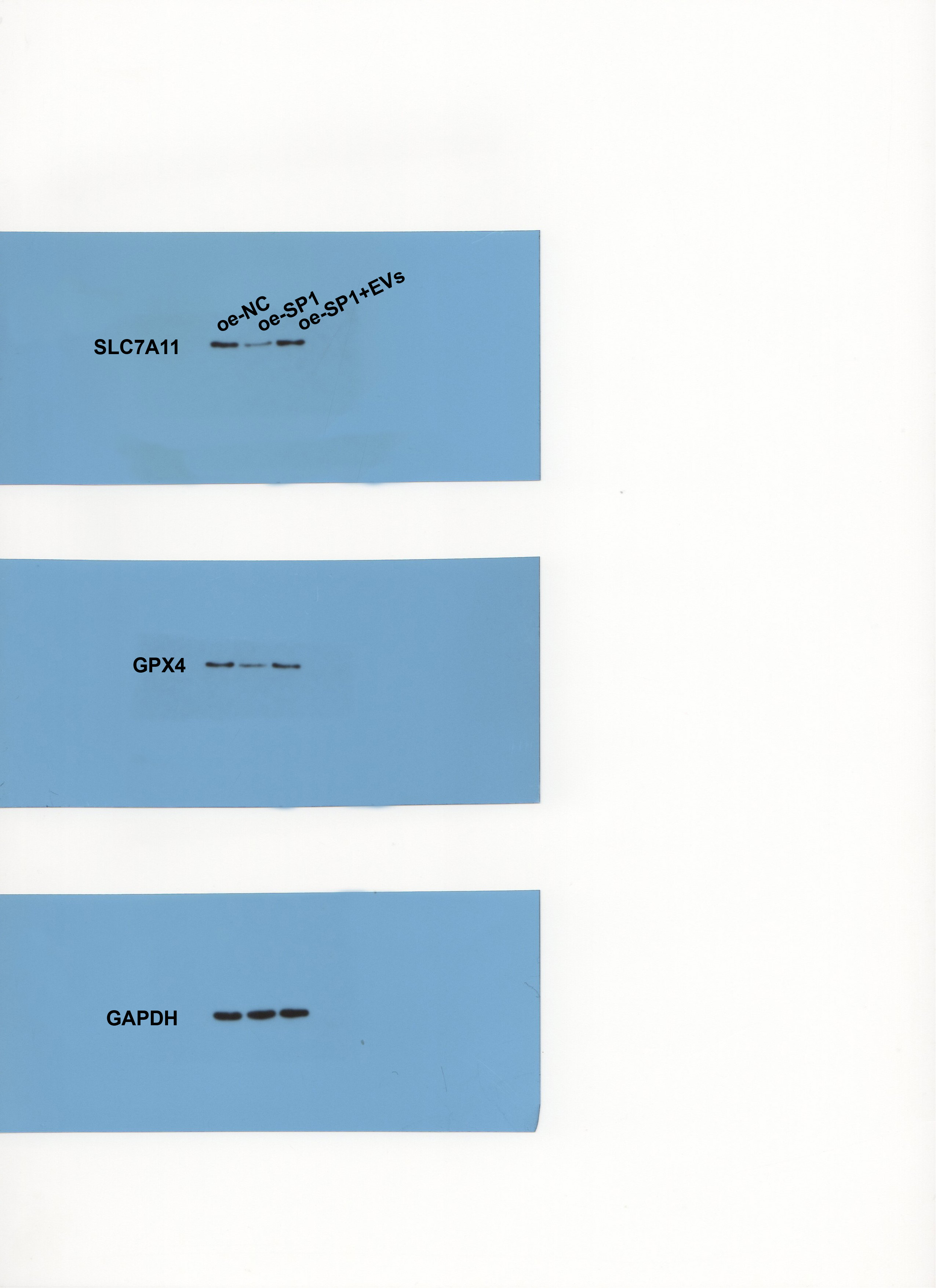

Supplement: Supplementary file 6 — WB 5E [file 41420_2021_610_MOESM6_ESM.jpg]

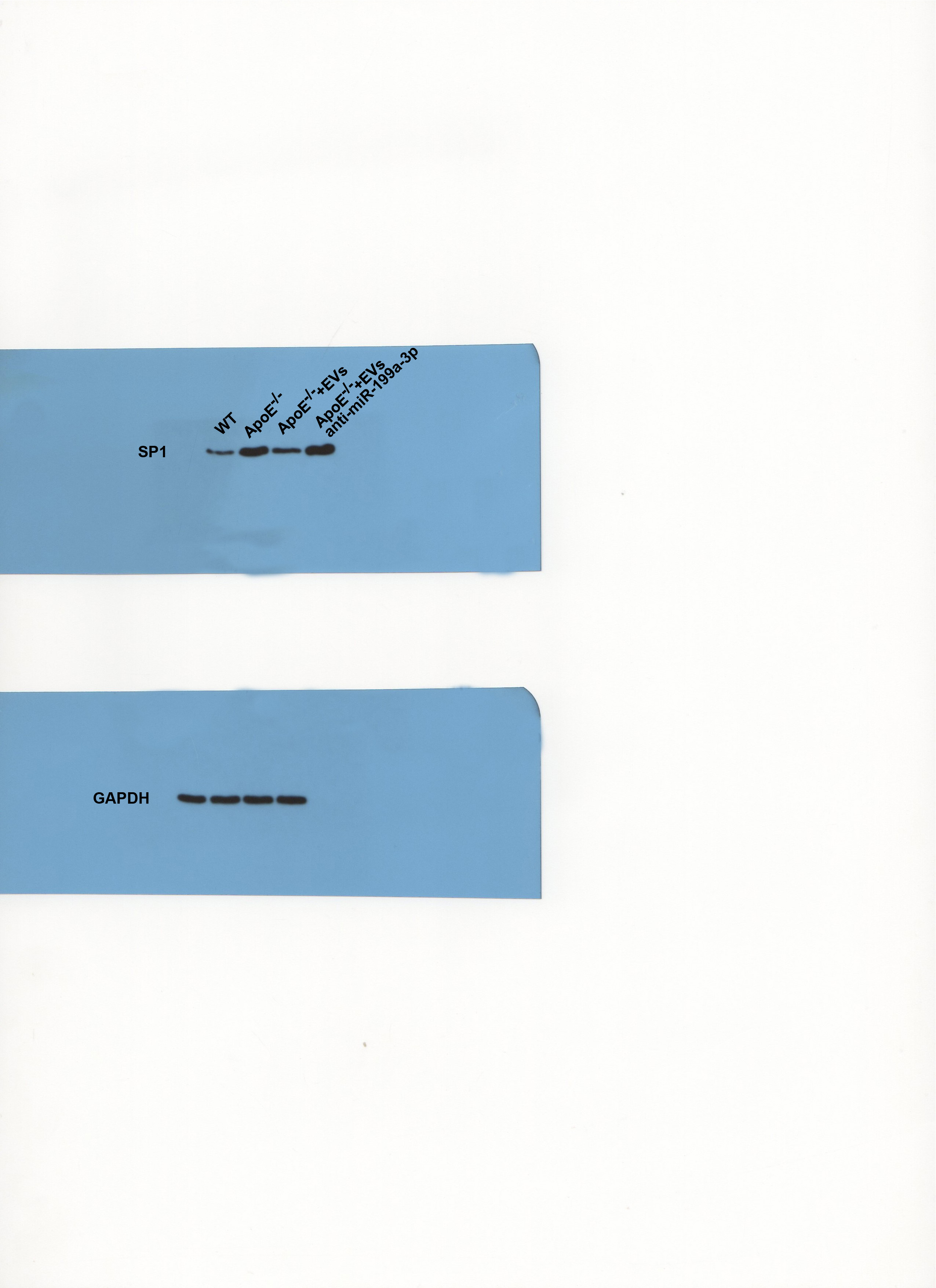

Supplement: Supplementary file 7 — WB 6B [file 41420_2021_610_MOESM7_ESM.jpg]

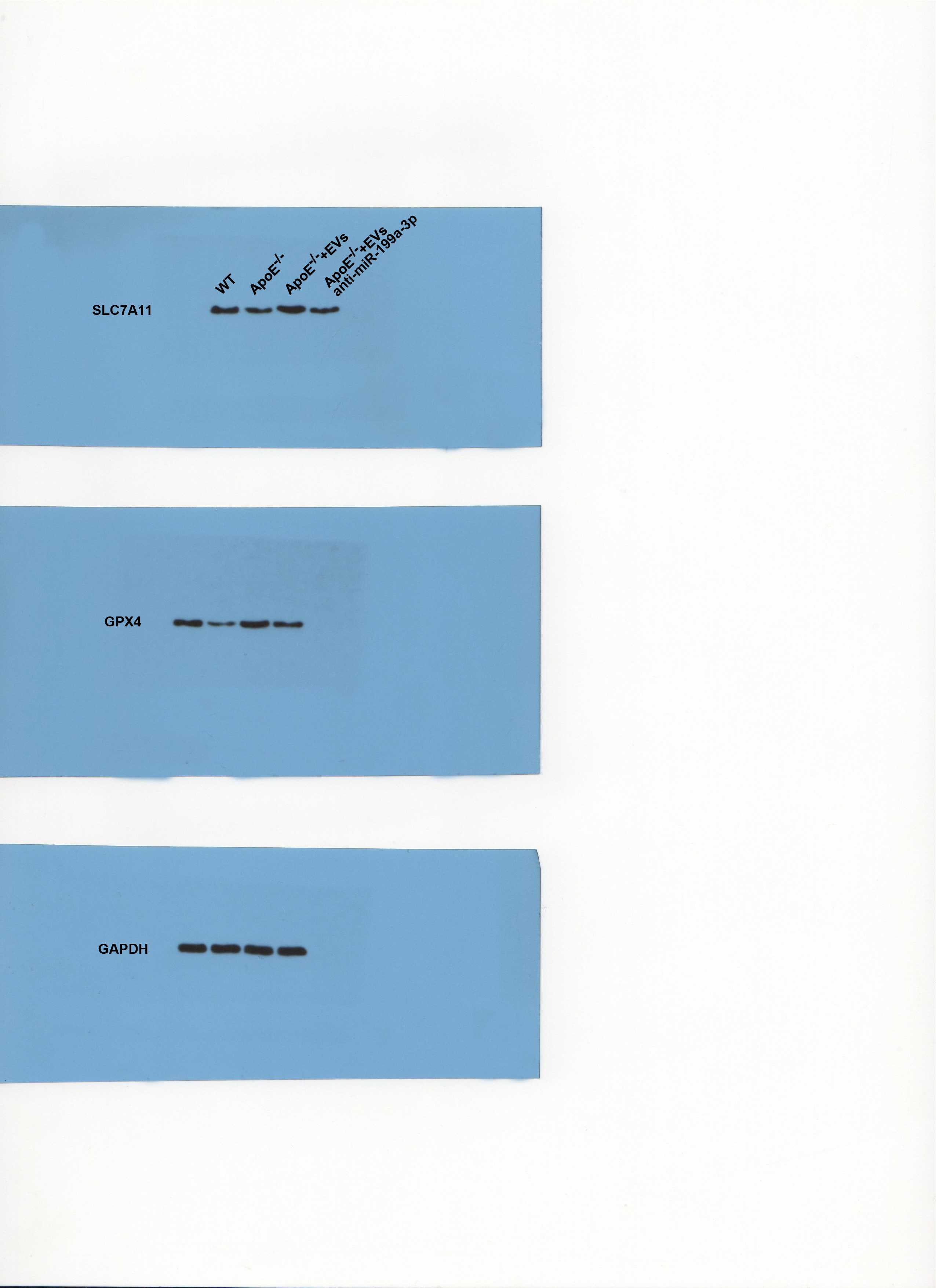

Supplement: Supplementary file 8 — WB 6H [file 41420_2021_610_MOESM8_ESM.jpg]
