# Supplementary material for: Effect of endothelial progenitor cell-derived extracellular vesicles on endothelial cell ferroptosis and atherosclerotic vascular endothelial injury
Source: Cell Death Discov. 2021 Sep 7;7:235. doi: 10.1038/s41420-021-00610-0 (PMC8423825; doi:10.1038/s41420-021-00610-0)
Supplement: Supplementary file 9 — Attribution of Authorship [file 41420_2021_610_MOESM9_ESM.pdf]

DECLARATION OF CONTRIBUTIONS TO ARTICLE

ADMC

Manuscript Number: CDDISCOVERY-21-1854

Journal Name: *Cell Death Discovery* (the 'Journal')

Proposed Title of the Contribution:

Effect of endothelial progenitor cell-derived extracellular vesicles on endothelial cell ferroptosis and atherosclerotic vascular endothelial injury (the 'Contribution)

Author(s):

Lin Li, Haining Wang, Jing Zhang, Xiao Chen, Zhongwang Zhang, Qiang Li (the 'Authors')

For all *CDDis*covery articles, each person named as an author in the published version must be able to show he or she has contributed substantially to the article.

Authorship credit should be based on 1) substantial contributions to conception and design, acquisition of data, or analysis and interpretation of data; 2) drafting the article or revising it critically for important intellectual content; and 3) final approval of the version to be published. Authors should meet conditions 1, 2 and 3.

Any person who cannot be shown to have made a substantial contribution to the article cannot be listed as an author in the final version. The name of any person who is deemed to have made a minor contribution can, however, appear in the Acknowledgments section of the article.

Please complete the table below to indicate the contributions of all named authors to the manuscript.

Author Full Name:	Specification of Contribution to the Manuscript:
Lin Li	contributed to the study concepts, study design, and definition of intellectual content
Haining Wang	contributed to the experimental studies and data acquisition
Jing Zhang	contributed to the literature research
Xiao Chen	contributed to the data analysis and statistical analysis
Zhongwang Zhang	contributed to the data analysis and statistical analysis.
Qiang Li	contributed to the study concepts, study design, and definition of intellectual content

Please complete the table below to indicate the contributions of all named authors to the figures.

Figure 1:

All the authors participated in the statistical drawing, typesetting and production of the experimental pictures and data analysis, and the examination and verification of the picture.

Figure 2:

All the authors participated in the statistical drawing, typesetting and production of the experimental pictures and data analysis, and the examination and verification of the picture.

Figure 3:

All the authors participated in the statistical drawing, typesetting and production of the experimental pictures and data analysis, and the examination and verification of the picture.

Figure 4:

All the authors participated in the statistical drawing, typesetting and production
of the experimental pictures and data analysis, and the examination and verificati
on
of the picture.

Figure 5:

All the authors participated in the statistical drawing, typesetting and production
of the experimental pictures and data analysis, and the examination and verificati
on
of the picture.

Figure 6:

All the authors participated in the statistical drawing, typesetting and production of the experimental pictures and data analysis, and the examination and verification of the picture.

Signed for and on behalf of the Author(s):	Print Name:	Date:
Qiang Li	Qiang Li	June 22, 2021
